# Development of an Intelligent Data-Driven System to Recommend Personalized Fashion Design Solutions

**DOI:** 10.3390/s21124239

**Published:** 2021-06-21

**Authors:** Shukla Sharma, Ludovic Koehl, Pascal Bruniaux, Xianyi Zeng, Zhujun Wang

**Affiliations:** 1 Laboratoire Génie et Matériaux Textile (GEMTEX), 59056 Roubaix, France; ludovic.koehl@ensait.fr (L.K.); pascal.bruniaux@ensait.fr (P.B.); xianyi.zeng@ensait.fr (X.Z.); hqxiaopan@126.com (Z.W.); 2 ENSAIT, 59056 Roubaix, France; 3 École Centrale de Lille, 59650 Lille, France; 4 College of Fashion and Design, Donghua University, Shanghai 200051, China; 5 School of Textile and Garment, Anhui Polytechnic University, Wuhu 241000, China

**Keywords:** fashion design recommendation, 3D virtual garment fitting, design knowledge base, intelligent algorithms

## Abstract

In the context of fashion/textile innovations towards Industry 4.0, a variety of digital technologies, such as 3D garment CAD, have been proposed to automate, optimize design and manufacturing processes in the organizations of involved enterprises and supply chains as well as services such as marketing and sales. However, the current digital solutions rarely deal with key elements used in the fashion industry, including professional knowledge, as well as fashion and functional requirements of the customer and their relations with product technical parameters. Especially, product design plays an essential role in the whole fashion supply chain and should be paid more attention to in the process of digitalization and intelligentization of fashion companies. In this context, we originally developed an interactive fashion and garment design system by systematically integrating a number of data-driven services of garment design recommendation, 3D virtual garment fitting visualization, design knowledge base, and design parameters adjustment. This system enables close interactions between the designer, consumer, and manufacturer around the virtual product corresponding to each design solution. In this way, the complexity of the product design process can drastically be reduced by directly integrating the consumer’s perception and professional designer’s knowledge into the garment computer-aided design (CAD) environment. Furthermore, for a specific consumer profile, the related computations (design solution recommendation and design parameters adjustment) are performed by using a number of intelligent algorithms (BIRCH, adaptive Random Forest algorithms, and association mining) and matching with a formalized design knowledge base. The proposed interactive design system has been implemented and then exposed through the REST API, for designing garments meeting the consumer’s personalized fashion requirements by repeatedly running the cycle of design recommendation—virtual garment fitting—online evaluation of designer and consumer—design parameters adjustment—design knowledge base creation, and updating. The effectiveness of the proposed system has been validated through a business case of personalized men’s shirt design.

## 1. Introduction

Industry 4.0 includes the Internet of Things, Smart Manufacturing, Cloud-based manufacturing and strictly follows the continuous integration of human in the manufacturing process to gain the continuous improvement [1]. Industry 4.0’s implementation in the fashion industry has given the rise to the advanced digital solution acceptance to automate the various activities of fashion supply chain at different levels. In previous few years, the fashion industry has witnessed an explosion in the number of digital solutions to build more personalized solutions. The big brands like Decathlon, Nike, and Adidas are using the innovative 3D virtual reality-based solutions for providing the highly customized garments. The research based on consumer survey done by Deloitte shows that customers are more likely to pay a high amount to get a more personalized experience for garment and accessories; price is not a barrier for them [2]. Further, following the literature of fashion recommendation system, the author has proposed and tested the Semantic Attribute Explainable Recommender Systems where this semantic-based system not only provide cloth recommendation to users, but also builds understanding about the various semantic attributes of the garment and the reason of specific semantic attribute [3]. “The state of fashion 2019”, Mckinsey’s survey report with US and EU purchasing managers, shows that 54% of participants mentioned that the proximity to consumer is becoming more important and 22% mentioned it will become more important in coming years [4]. Other fashion recommendation models, which are based on the traditional recommendation filtering techniques. These techniques are as follows:

- Content-based filtering (CBF): The CBF technique parses an item’s description set to analyze the user’s profile by matching the mentioned description set into their profile. Previously, authors have developed the clothing recommendation model by analyzing the content based image and fashion keyword library for providing best out of garment knowledge base [5,6,7].
- Collaborative filtering (CF): Recommendations are usually generated by evaluating previous feedback so that set of similar users and items can be identified. Authors have applied the CF filtering technique to build fashion awareness personalized ranking function by evaluating user’s feedback [8,9]. Another system where an author presented the visually-aware recommendation system based on the implicit feedback and visual signals in the system like product images is linked to the recommendation task to improve the system by providing the matching styles of garments [10].
- Hybrid filtering (HF): Two or more filtering techniques are utilized to combine their benefits. The HF technique has been widely used in e-shopping platforms. Furthermore, HF resolves the well-known problem of cold-start, content over specialization of collaborative, and content-based filtering systems [11,12]. Further, in the literature, an advanced hybrid recommendation system where the authors of [13] presented a Dual hybrid recommendation systems that involved user ratings and user/item features being combined to generate the recommendation.
- Social media-based recommendation system: In addition to the traditional filtering techniques, social media-based recommendation systems have been developed by tracking the user’s implicit interaction over the social media platforms. Authors have mentioned in the literature that such systems bind the social networking activities with the user’s historical data and generates more accurate, trending, and useful suggestions [14,15]. Previous research work has clearly shown the integration of similar social media activities of users have the similar tendency to choose the items [16] and such system helps to track down the wearing habits of community.
- Design-oriented recommendation system: The existing literature has also shown many fashion design-oriented personalized recommendation systems. The authors of [17] developed a system to create the apparel item set by evaluating the attribute coordination of a different set of apparels based on a knowledge base using the Takagi–Sugeno fuzzy neural network (TSFNN), solving an inevitable decision-making problem. Another system used a interactive genetic algorithm-based design model to give functionality to the users to sketch their apparel with a user-friendly interface, enhancing the ease of designing own clothes without dealing with the garment pattern’s complicated parameters [18].

As per the literature of fashion recommendation systems, we rarely found a system considering below mentioned issues to build the system. These points are as follows:

1. The current designing tools or digital platforms are usually separated from each other because of their separate user base, i.e., some digital tools, like 3D computer-aided design (CAD), are only for designers and web-based design tools are for garment buyers. A lack of integration between these user bases hinders the efficiency of these tools in the whole design and manufacturing processes.
2. Being a traditional industrial sector, much professional knowledge related to fashion products like technicality behind the design of product, designer’s experience, and perception plays a key role in fashion industry. However, this knowledge is rarely involved in the current computational tools where the garment buyer is the common user base.
3. The relationship between different factors, especially between human perception on the finished product and technical parameters, is usually complex and cannot be mastered easily.

The proposed data-driven interactive design system brings four original contributions in comparison with the existing literature:

1. User (garment buyers) knowledge base integration with a design knowledge base (professional designer’s activity).
2. 3D ease knowledge base integration with 3D CAD tool using the proposed service architecture.
3. Adjustment of proposed design solution based on the feedback from 3D garment fitting evaluation, in order to generate personalized design solution.
4. Existing fashion recommendation systems where most of the solutions work with either the design elements, fitting, or ease preferences according to the garment buyers or professional designers, the proposed system originally combines these three factors of garment design process (design elements, fitting, and ease) preferences according to the professional designers as well as garment buyers’ point of view. This system easily enables more complete design solution where garment buyers and designers can easily evaluate the product and their preferences can be saved into system.

**Figure 1:**
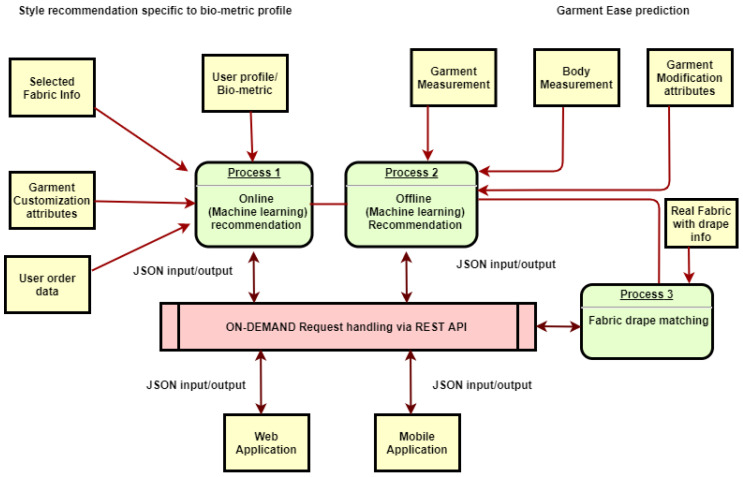
Data-driven service architecture.

**Figure 2:**
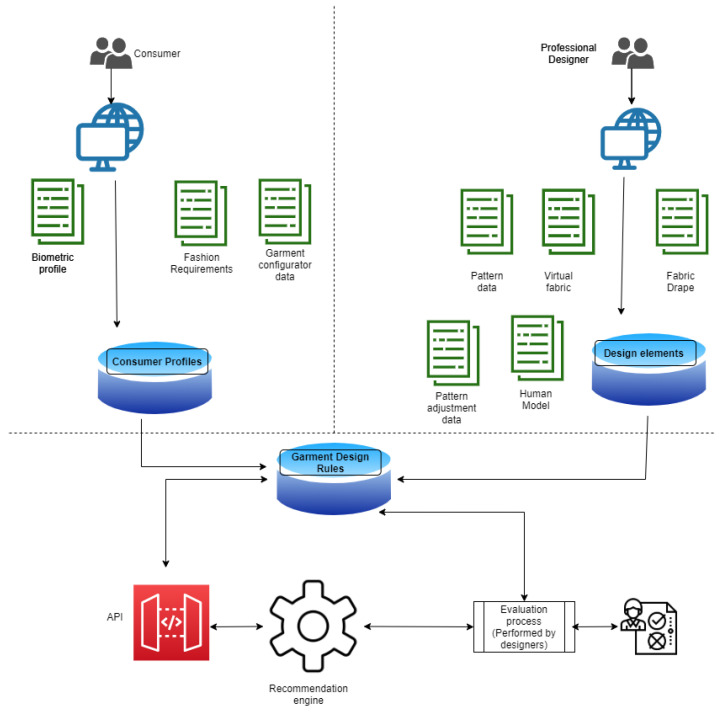
The structure of the proposed garment knowledge base.

The general principles of the proposed recommendation service can be applied to all kinds of garments. The proposed service specifically targets the online garment configurator of the men’s formal made-to-measure shirt knowledge base, where recommendations and suggestion are generated based on the similar biometric profile. It will create a professional designer’s knowledge base in order to help the designer to design the most relevant customized garment according to the consumer’s biometric data describing his/her body shape and desired garment fashion style selected. In the related computational models, the techniques of incremental machine learning, including BIRCH (), incremental clustering, and Adaptive random forest classifier, have been used to produce the best real-time recommendations. The proposed system architecture has been followed to build data-driven interactive design system. A complete proposed service architecture can be seen in Figure 1. Section 2 gives an overview of the design knowledge base creation steps with the help of Figure 2, where a web-based garment configurator has been used to acquire the relevant data of shirt customization, the biometric profile, customized attribute, and a detailed view of the data-driven service architecture. Section 3 gives an detailed description of each step’s input and output. Section 4 describes the modeling of the data-driven service using data mining algorithms, incremental clustering, incremental classification, association mining, and the radial basis function approximation method. Section 5 presents the service architecture validation, and Section 6 presents the conclusion.

## 2. Garment Design Knowledge Base Creation

Garment design knowledge base creation includes the following dataset:

1. Consumer profile dataset (biometric parameters related to customized garment, fashion requirements, and garment configurator data related to garment types to be selected by consumers).
2. Garment design process dataset (garment patterns, parameters of real and virtual (digital) fabrics with various colors, 3D human models adapted to the target population, and pattern adjustment parameters).

Consumer profile data and the predefined design attributes of garments(men’s formal shirts) have been collected from an online e-shopping platform. The 3D CAD tool has been utilized to collect the data associated with the designer’s activity of the customized garment creation process. Then, the association rules over the collected data generated by exploiting the relations between consumer data and data on design attributes of shirt like the fabric collar, cuff, etc., so that the best combination of garment design elements with higher confidence level can be determined as insight for the garment buyers as well as the designers. The structure of the proposed garment knowledge base is shown in Figure 2.

## 3. Flow Chart of Proposed Data-Driven Interactive Design System Architecture

**Figure 3:**
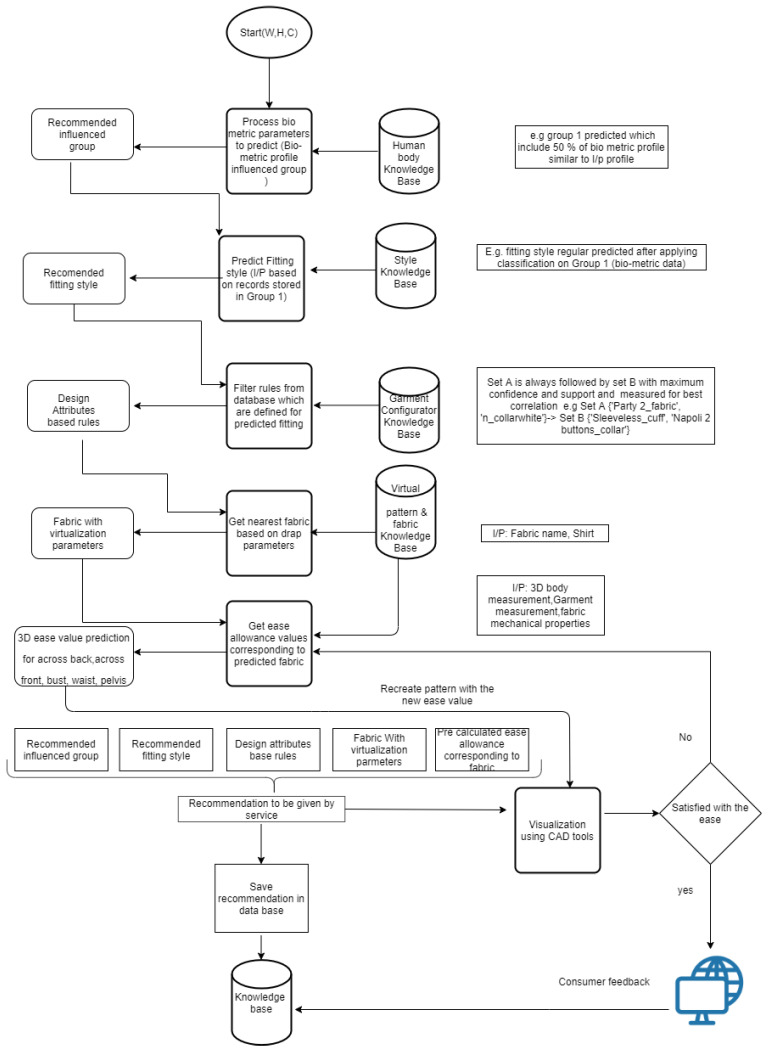
The flow chart of the data-driven interactive design system.

The proposed system (see Figure 3) aims at recommending the most relevant garment design solution for the specific morphology by supplying the biometric parameters (weight, height, collar size), and these attributes are denoted as W, H, and C, respectively, in Figure 3. The number of biometric parameters strongly depends on the type of garment and the customized design elements provided by the fashion e-shopping platform. The attribute collection for men’s formal shirts is limited to the data provided by the fashion company. The proposed interactive design system has been composed of five operational modules, which results (influenced by body shape group, garment fitting style, design attribute-based rules, fabric parameters, and ease allowance) in prediction and finally constitutes together to give the final design solution. The following points give the brief of these operational modules:

1. Prediction of most relevant body shape group based on the user’s biometric profile. The similarity was found using the incremental clustering BIRCH algorithm.
2. Prediction of the most relevant garment fitting style adapted to the identified human model, which is related to body measurements. Incremental classification model trained using adaptive random forest classifier to get the prediction.
3. Identification of the most relevant garment style attribute combinations by filtering the association rules knowledge base. The association rule knowledge base has been created using the FP-growth algorithm.
4. Identification of the most relevant fabric by finding the similarity between the drape image profile of real fabric and existing virtual fabric library, which is associated with the 3D CAD tool and Kd-tree algorithm has been utilized to find the nearest fabric from the existing knowledge base. This module will help to build insight in advance for manufactures and designers to judge new fabric’s performance based on its similarity with the existing similar fabric’s performance.
5. Radial basis function neural network (RBFNN) has been utilized to build the 3D ease prediction model, which is trained over the body measurements, shirt pattern measurements, and fabric mechanical properties. This module will help the new designers to recreate the new shirt pattern.

## 4. Modeling of Data-Driven Interactive Design System

The model building process divided in five consecutive steps to establish the proposed system architecture. All steps have been shown in Figure 1. This section provides the detailed description of data mining techniques (clustering, classification, and association mining) and radial basis function approximation method to build the prediction model.

### 4.1. First Step: Prediction Model to Find Most Relevant Biometric Profile

#### 4.1.1. Balanced Iterative Reducing and Clustering Using Hierarchies Clustering Theory

BIRCH stands for Balanced Iterative Reducing and Clustering using Hierarchies. BIRCH is an unsupervised hierarchical clustering data mining algorithm. The BIRCH algorithm usually applied to build dynamic and incremental clustering model and works on multidimensional metric data points; it handles a large data set with superior time complexity and space efficiency [19]. The BIRCH clustering algorithm consists two steps to extract clusters from data:

- Build the CF Tree: The first step is building the CF Tree and loading data into a cluster feature tree (CF Tree). The CF Tree represents or keeps data in a compressed form in memory. The BIRCH algorithm becomes highly efficient by using summary statistics for minimizing large data sets. The CF Tree is built with CFs and each CF is composed of three summary statistics [19]:
  - The N represents the number of data points in the cluster $\left\{\begin{array}{c} \vec{X_{i}} \end{array}\right\}$ where *i* = 1, 2, …, N.
  - The linear sum is the sum of individual data points and helps to measure the location of the cluster where $\left\{\begin{array}{c} \vec{X_{i}} \end{array}\right\}$ is a cluster.
    (1) $$\vec{LS}=\sum_{i=1}^{N}\vec{X_{i}}$$
  - The squared sum is the sum of squared data points and helps to measure the spread of the cluster, where $\left\{\begin{array}{c} \vec{X_{i}} \end{array}\right\}$ is a cluster.
    (2) $$SS=\sum_{i=1}^{N}{\left(\vec{X_{i}}\right)}^{2}$$
  A cluster feature tree is a tree structure composed of CFs. A CF tree represents a compressed form of data, preserving any structure in the data. A CF tree has the following parameters:
  - Branching Factor B. It determines the maximum children allowed for a non-leaf node.
  - Threshold. It gives the upper limit to the radius of a cluster in a leaf node.
  - Number of Entities in a leaf Node L.
- Global Clustering: The second step is clustering the sub-clusters. After the creation of the CF Tree, the existing clustering algorithm on the CF Tree Leaf nodes(sub-clusters) is applied to combine sub-cluster into clusters.

#### 4.1.2. Building Prediction Model by Applying BIRCH Incremental Clustering Algorithm

**Figure 4:**
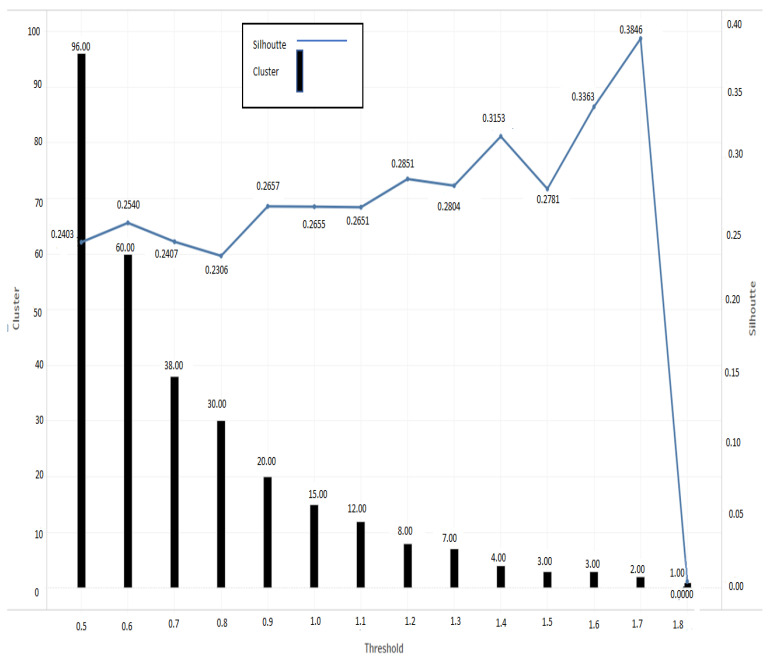
Cluster evaluation corresponding to different threshold.

**Figure 5:**
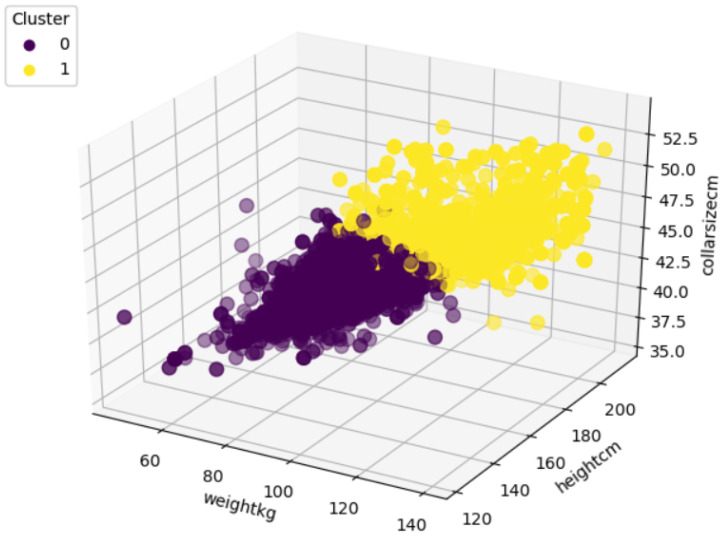
Cluster formation with silhouette score 0.3846.

The customized men’s shirt order dataset contains 5291 records, which includes both males and females. The order dataset has 4898 records for customized men’s shirts and 393 women’s shirts data. Therefore, in total 4898 records have been utilized to train the model. In this step, we trained an unsupervised incremental clustering model, permitting a biometric profile corresponding to the chosen customized garment (men’s shirt) to be classified according to the user biometric profile. First, we acquired the data from fashion e-shopping digital platform, which are specifically dedicated for men’s customized shirt. A three-dimensional input vector (height, weight, collar size) was extracted from the user’s biometric profile data. The implementation steps of the birch algorithm using the men’s shirt dataset are as follows:

- First, selection of the appropriate threshold T value, where T is the upper limit to the radius of BIRCH cluster in a leaf node and radius is obtained by merging the new samples.
- The best value for threshold T has been selected based on the initial collected data and analyzing the silhouette score.
- Figure 4 shows silhouette score for choosing the best threshold value to setup initial model and to find the cluster of biometric profiles. silhouette score value ranges between (−1,1).
- The silhouette score was calculated to evaluate the cluster’s formation corresponding to threshold value. In Figure 4, it can be clearly seen that threshold value 1.7 predicts 2 cluster with maximum silhouette score 0.3846 which indicates better cluster formation. Therefore, threshold value 1.7 has chosen to set up biometric profile prediction model.
- The BIRCH model created a total of two clusters from the initial dataset corresponding to the threshold value 1.7, and its formation and total record count are shown in Figure 5 and Table 1, respectively.

### 4.2. Second Step: Prediction Model to Find Most Relevant Fitting Style

#### 4.2.1. Adaptive Random Forest (ARF) Classification

Adaptive random forest (ARF) classifier is a streaming classifier. ARF is an updated version of random forest classifier. ARF classifier has been utilized well to build the classification model over streaming data. Also, to build real time data mining classifier model. Contrarily traditional random forest classifier needs to be retrained fully by merging new set of data to the existing dataset or just override the previously trained version with new one. Previous research work has shown the significant use of ARF classifier, which is described as follows [20]:

- Random forest classifier requires multiple passes over input data and it becomes infeasible if classifier is handling streaming data.
- Random forest to streaming data application requires an online bootstrap aggregating process; and limiting each leaf split decision to a subset of feature.
- ARF uses the drift detection method to cope with evolving data stream.
- Once the drift is detected ARF uses a threshold value to detect the warning and resultant create a background tree that a trained along the ensemble without affecting the existing ensemble’s prediction. Drift detection raises warning signal, which then replaces the respective background tree.
- In ARF votes are weighted using the test then train accuracy method.

#### 4.2.2. Building Shirt Fitting Style Prediction Model

In the previous step, we segregated the user’s profile by using the biometric parameters as per the collected data. Further, in step 2, N ARF classifier models were trained where N is the number of clusters found in step 1. The existing dataset was separated into two clusters by applying the unsupervised BIRCH clustering algorithm. This model has been used to find the most relevant fitting style to a specific biometric profile of customized garment; here, men’s formal shirt has been taken as a customized garment. First, we set up the ARF classifier by supplying input vector [X, Y] where X consist of three biometric parameters (height, weight, collar size) and Y contains fitting type (Super Slim Fit, Comfort fit, Regular). Table 1 shows the three different types of fitting style with its count value. Second, we performed an interleaved test, then trained the method to evaluate the ARF classification model [21]. The incremental classification prediction model cannot be evaluated by applying cross-validation measures, unlike the static and batch learning classification. The prequential evaluation is sequential analysis where the sample size is not fixed initially and the model is evaluated as new data is collected. The prequential evaluation first uses each instance to test the model and then train the model. Table 2 shows the mean performance using accuracy and kappa score measures for individual classifiers trained over sub-datasets processed in step 1. The kappa score ranges between 1 and −1, where 1 implies perfect agreement and values less than 1 indicates less than perfect agreement [22].

### 4.3. Third Step: Prediction for Most Relevant Fitting Style Rules

The online garment configuration tool on the e-shopping platform gives each and every attribute of the garment and helps the buyer to make their own version of garment. Therefore, here a question arises as to how the designer can know the buyer’s preferences and the pattern of selecting style attributes. It is necessary to explore the relationship between the styling attributes and the buyer’s styling attribute selection behaviour as well as getting the right product at the right time. Inevitably, profitability of the business can be raised by making the satisfactory product on time for consumers [23]. In this step, model builds the best combination of customized shirt attributes for the predicted fitting style. Set of attributes for a specific fitting style (Super Slim Fit, Comfort fit, Regular) calculated in this step applying association mining algorithm.

#### 4.3.1. Association Mining

Association rule mining is a data mining technique that is used to study attributes or characteristics that are associated. Traditionally, association rule mining algorithms extracts the frequent feature or item sets. It uncovers the rules to measure the relationship between two or more attributes. This module works in two stages to process the data:

1. Finding frequent attribute sets in data within the range of a defined support count value.
2. Using the frequent item sets to generate the association rules. Only frequent attribute sets that fulfill the minimum threshold value are used to generate the association rules.
3. Then, filtering the rules based on the threshold value assigned for support, confidence, and lift.

Let $I\;=\;\left\{\begin{array}{c} i_{1},i_{2},i_{3},....,i_{d} \end{array}\right\}$ be the set of all features of customized garment dataset and $T\;=\;\left\{\begin{array}{c} t_{1},t_{2},t_{3},....,t_{N} \end{array}\right\}$, where each transaction *t_i_* contains subset of features chosen from *I*. Let *A*,*B* be sets of items; an association rule is a derivation of the form A⇒B, where A⊂I, B⊂I, and where A and B are disjoint itemsets, that is, A∩B=∅. The strength of extracted rules can be measured by calculating the *support* and *confidence* [24]:

- *Support* (s) determines how often rule is applicable to a given dataset.
  (3) s=P(A∩B)
- *Confidence* (c) of the association rule A⇒B is the measure of accuracy of rule, which is determined as the percentage of transaction in dataset containing A that also contains B.
  (4) $$c=P\left(B|A\right)=\frac{P(A\cap B)}{P(A)}$$
- *Lift* (l) computes the ratio between the rule’s confidence and the support of the itemsets in the rule consequent [25].
  (5) $$l=\frac{P(A\cap B)}{P(A)P(B)}$$
  The authors of [25] have interpreted the lift measure as follows:
  (6) $$Lift=\left\{\begin{array}{cc} =1 & \mathrm{if}\;A\;\mathrm{and}\;B\;\mathrm{are}\;\mathrm{independent};\\ >1 & \mathrm{if}\;A\;\mathrm{and}\;B\;\mathrm{are}\;\mathrm{positively}\;\mathrm{correlated}\\ <1 & \mathrm{if}\;A\;\mathrm{and}\;B\;\mathrm{are}\;\mathrm{negatively}\;\mathrm{correlated} \end{array}\right.$$

#### 4.3.2. Implementation of Association Rule Mining to Extract Rules for Shirt Feature Sets

- Frequent item set generation process finds all item sets which satisfy the minimum support threshold value.
- The proposed system generates n tables for each cluster. Let proposed system receives different fitting type element $f_{1},f_{2},f_{3},\ldots ,f_{s}$ with repetition. Let *d* be the number of distinct elements namely $d=|\{f_{1},f_{2},f_{3},\ldots ,f_{s}\}|$, and let these elements be $\left\{g_{1},g_{2},\ldots ,g_{n}\right\}$. Finally, let $c_{j}$ be the count of $g_{j}$. Thus $\sum_{i=1}^{n}c_{i}$ represents total number of frequent pattern set tables generated by step 3. Similarly, in continuation, a similar number of table schema corresponding to each frequent pattern in the relational database management system are created via a service.
- From the frequent item sets generated in previous step, the rule generation step extracts the rules satisfying minimum support and confidence condition.

To generate frequent item sets from the transaction database, we have used the FP-growth algorithm. The FP-growth algorithm has been used successfully in large transaction database, because of the use of compact data structure FP-tree to get frequent item sets efficiently and quickly without generating the candidate sets explicitly unlike an a priori algorithm [26]. The following steps have been followed to generate the frequent feature sets of the customized shirts transaction database.

1. Create frequent item sets by applying FP-growth to the shirt’s feature dataset with minimum support value 0.1 to cover most of the features in item sets.
2. Read data from database *D*, which contains all the records of shirt’s feature transaction data. Corresponding to each fitting type (Comfort Fit, Regular, Super slim fit), which is considered as labels for the classification model let *C* contains distinct class; the FP-growth algorithm is applied to get the frequent item set corresponding to each fitting class.
3. Using the frequent item sets, the system generates association rules satisfying the minimum support and minimum confidence (0.8) conditions.
4. Table 3 and Table 4 show the statistics for each fitting class rules collection in cluster 1 and cluster 2.
5. Rules extracted from database by matching the fabric name in antecedents to get the best shirt feature combination.
6. Association rule’s A⇒B usefulness and positive correlation has been measured using *lift l, where l>1*, which indicates positive correlation between the antecedent and consequent item or feature set.
7. For a sample case, cluster 1 has been predicted for input vector weight,height,collarsize, let these elements be w=80,h=190,c=42, cluster 1 has been predicted for the given input vector and step 2 has predicted fitting type Regular, and step 3 has read data from association rules table for regular fitting. Table 4 shows the count of association rule table corresponding to each available fitting type in the system. Corresponding to regular fitting as it has been predicted in step 2 there has been 39 rules filtered from the knowledge base, which are then further used to build the best shirt feature combination for regular fitting. Then, the rules are further filtered by matching antecedents with a specific shirt feature. Let us say, the rules related to cuff extracted to know, which other feature of shirt “goes together”, like collar style, fabric type, and Collar white. Furthermore, evaluation metric confidence and lift utilized to get the strongest rules for the prediction, and only rules with confidence value greater than 0.80 and lift value greater than 1 considered. Matching rules for cuff are shown in Table 5.
8. computational time for the complete step starting from the finding the suitable group for user as per his biometric profile then most relevant fitting style and then the combination of styling attribute combination for the predicted fitting style from the association rules is 558 ms per request.

### 4.4. Fourth Step: Acquisition of Real Fabric Data and Knowledge Base Integration

In the literature of CAD clothing design tools, the fabric drape has been considered as an essential and key parameter, and previous research work has shown that mechanical properties are highly correlated to the drape of the fabric [27]. If garment and fabric simulations are closer to the realistic version of garment and fabric, then this digital information can help fabric and garment manufacturers to take an adequate action before actual production. It has also been noticed in the literature that similar size garments with similar styles made from different fabrics have shown easy variations [28]. Correct drape measures and detailed description for the virtual clothing makes it possible for designers that how good a garment will look on the avatar. Furthermore, drape is a great indicator to know the garment conformity and body contour. Therefore, the proposed system has used a 3D CAD tool to build the virtual fabric knowledge base by collecting parametric information of complex fabric simulation structure. In the literature, different fabrics were compared using their mechanical properties and how these properties affect the fabric drape stiffness, garment ease, and fit of the virtual garment [29]. Consequently, to build collaborated garment design knowledge base virtual fabric’s and real fabric’s drape image profile created and analyzed by comparing the drape contour of fabric. Therefore, the existing insights for similar drape fabric can be used for the new fabric at designing phase of the virtual garment. Thus, to achieve the integrated solution for designers and consumers, fabric drape data need to be considered as an essential part. Moreover, although existing CAD tools for virtual garment creation process contain a huge library for virtual fabrics of different types, we have used the fabric drape image analysis process to utilize the correlation between the fabric drape and its technical parameters in order to find the existing virtual fabric from the database, which can be compared to the real fabric based on the drape image profile. The following parameters have been considered as per the fabric drape image profile literature:

- Amplitude (A) is half the difference between maximum and minimum radii. It gives the depth of the drape profile with respect to the radius of draped fabric image [30]: AA in below equation represents average amplitude
  (7) $$AA=\frac{1}{n}\sum_{i=1}^{n}\frac{p_{i}-v_{i}}{2}$$
  where $p_{i}$ is the maximum radius of the draped fabric image profile, $v_{1}$ is the minimum radius of the draped fabric image profile.
- Average distance: average distance from zero or mean of contour
  (8) $$AD=\frac{1}{n}\sum_{i=1}^{n}\frac{p_{i}+v_{i}}{2}$$
- maximum peak: $p_{max}$ is the maximum radius of peak from center.
- maximum valley: $v_{max}$ is the maximum radius of valley from center.
- N is the number of peaks in the draped shadow.

In this step, the real drape contour image integrated to the virtual fabric library by creating the drape profile using polar coordinate technique. The polar coordinate method has been used widely and successfully to analyze fabric drape in a low-stress mechanical way. Integration of the virtual fabric knowledge base could help the designers; therefore, new real fabric integration into the knowledge base and finding fabric with similar drape can be utilized to draw the insight for new fabric before starting the production process. The proposed data-driven service can be used by designers and technologists to get insights of new fabric by correlating with existing fabrics and garment fitting style knowledge base.

The following steps have been followed for building the virtual fabric knowledge base using fabric drape image as follows:

- The Drape profile for each virtual fabric was created using the polar coordinate technique and extracted parametric information was added to the knowledge base; similarly, for the real fabric drape image profile and respective parameters added, average amplitude, average distance, maximum peak, minimum valley, and number of nodes of drape contour image.
- The system builds insights for new fabric from the existing virtual fabric knowledge base. Unsupervised kd-tree algorithm was trained over the fabric image profile parameters to get the nearest fabrics from the system.

### 4.5. Fifth Step: Acquisition of 3D Ease Allowance Parametric Information

Further, in this section, literature related to the garment ease and its automation process is presented and followed to build the prediction model to predict the 3D ease value of key body parts. We found various studies for the garment ease calculation process for virtual garment to speed up the garment making process. One paper proposed a methodology to define, quantify, and control the ease allowance in the 3D virtual CAD tool by defining a template of 3D ease allowance for personalized garments [31]. However, it has been mentioned in the proposed study that to achieve the baseline template to automate the ease allowance, the whole process of garment design needs to repeated. Therefore, that the ease allowance template using consumer morphology can be achieved, and then once it is achieved it can be automatically utilized for the garment customization in the 3D CAD tool environment [31]. Another smart and efficient pattern making technique introduced by the author, where the proposed method has been proved robust and well suited for loose and tight-fitting garments, also collectively considered garment ease allowance, fabric elasticity, and draping to propose a versatile pattern making approach in a 3D environment [32]. In the literature, a method of reconstructing 3D individual body shape for a skirt in a computerized pattern making system has been proposed where the author has created unique body shape by utilizing ease allowance values for pattern making [33]. In the literature mentioned above, we found that advanced 3D CAD tools always require an expert’s knowledge, activity, and supervision to achieve the best solution by doing the repetitive action. In this context, we have worked to automate the process of getting the ease value for the garment using the radial basis function approximation method. The proposed 3D ease prediction model effectively can reduce the professional designer’s intervention and can help the new designers to learn from the established prediction model based on the professional designer’s activity. Proposed automated process of ease allowance prediction model based on the past action of professional designers. So that the garment pattern making process could become easier and quick for the new designers. Furthermore, the fabric insights based on the ease value can be generated for better choice of fabric.

#### 4.5.1. General Scheme

**Figure 6:**
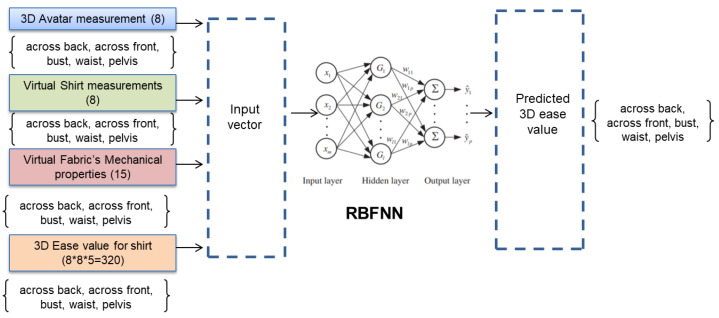
Architecture of ease prediction model.

The general scheme of 3D ease allowance prediction model is created based on the Figure 6 and used radial basis function neural network(RBFNN) architecture to build prediction model. Following steps to build the 3D ease prediction model are as follows:

- A 3D garment ease prediction model has been developed using the RBFNN model. First input set to the model is key body parts (across back, across front, bust, waist, pelvis) of 3D avatar extracted from the lectra 3D CAD tool have been used, second input set to the model is garment measurement corresponding to each key body part of the actual garment, the third input set is the mechanical properties of fabric, and the output set contains ease allowance data corresponding to key body parts (across back, across front, bust, waist, pelvis).
- The proposed model tested and validated by splitting the initial data set into two subsets: First subset of data have been used to train RBFNN, second subset of data have been used to validate the trained model to evaluate the generalization ability of model.

#### 4.5.2. Learning Data Acquisition

Steps to build the knowledge base for digital garment creation process is as follows.

- **Step 1:** Eight different body measurements were considered to construct 3D mannequins using 3D lectra CAD tool and the key body parts measurements are collected and shown in Table 6.
- **Step 2:** Standard men’s shirt size chart shown in Table 7 has been utilized to construct the shirt pattern for the 3D mannequins constructed in step 1.
- **Step 3:** Each shirt pattern then further utilized to create the virtual shirt for the 3D avatar constructed in step 1 and the final shirt garment measurement data obtained as shown in Table 8.
- **Step 4:** Each and every virtual shirt created in 3D tool lectra then further tested against the five different fabrics with different fiber compositions and its mechanical properties. Detailed description of chosen fabric has displayed in Table 9, Table 10 and Table 11.
- **Step 5:** After the construction of virtual shirts ease data collected for key body parts of shirt (across back, across front, bust, waist, and pelvis). Each key body part’s five ease values obtained corresponding to 5 chosen fabrics to see the influence of fabric on the garment ease.
- **Step 6:** All the 8 human body measurements, 8 different shirt sizes, and 5 fabrics with different fiber composition are utilized to evaluate the 3D ease of virtual shirt. This experiment resulted to 320 records to make the knowledge base, obtained records then further utilized to implement 3D ease non linear functional approximation prediction model.

#### 4.5.3. Learning Data Formalization

Steps to train the prediction model has been formalized as follows: Let $F=\{f_{1},f_{2},.....f_{n}\}$ where *n* = 5 are involved fabric set. Let $F_{j}P=\left\{f_{j}p_{1},f_{j}p_{2},.....f_{j}p_{m}\right\}$, where m = 15 and $f_{j}p_{m}$ is the $m^{th}$ mechanical property of fabric $f_{j}$ where $f_{j}\left(j\epsilon \left\{1,2\ldots ,n\right\}\right)$. Let $P=\{p_{1},p_{2},\ldots ,p_{n}\}$, where *n* = 8 be a set of male body measurements involved. Let $P_{i}BPM=\left\{p_{i}bpm_{1},\;p_{i}bpm_{2},\;\ldots ,\;p_{i}bpm_{q}\right\}$, where q = 5 is the involved key body parts measurement set and values are extracted from 3D avatar of $p_{i}$ where $p_{i}\left(i\epsilon \left\{1,\;2\;\ldots ,\;n\right\}\right)$. Let $P_{i}BPGM=\left\{p_{i}bpgm_{1},p_{i}bpgm_{2},\ldots ,p_{i}bpgm_{q}\right\}$, where q = 5 is the vector of involved key body parts garment measurements extracted from 3D virtual garment of $p_{i}$ where $p_{i}\left(i\epsilon \left\{1,2\ldots ,n\right\}\right)$. Let $P_{i}E=\left\{p_{i}e_{1},p_{i}e_{2},\ldots ,p_{i}e_{q}\right\}$, where q = 5 is vector of involved key body parts to measure the ease allowance for $i_{th}$ person P $p_{i}$ where $p_{i}\left(i\epsilon \left\{1,2\ldots ,n\right\}\right)$.

#### 4.5.4. Implementation of Radial Basis Function Neural Network

**Figure 7:**
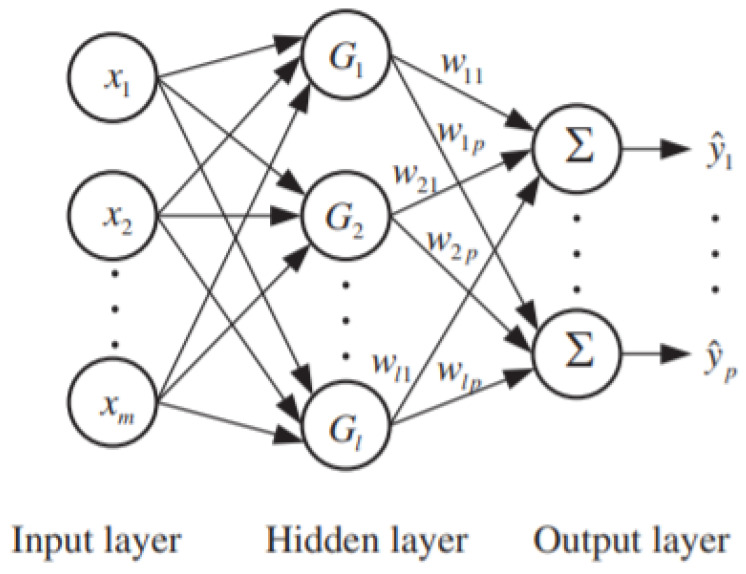
Architecture of RBFNN [37].

The radial basis function neural network (RBFNN) is a feedforward neural network model with proven universal approximation ability with no local minima problem [34]. RBFNN is a neural network that uses radial basis functions as activation functions. The RBFNN architecture contains three layered feedforward neural network [35,36]. The first layer of the RBFNN receives input and distributes the input signal to hidden layer and neuron in the input layer, which corresponds to each predictor variable; it has a second layer which has a hidden layer and uses radial basic function functions; the means in each neuron in the hidden layer consist of a radial basic function like Gaussian function at the end finally third layer makes the output layer and has a weighted sum of outputs from the hidden layer. Figure 7 shows general architecture of radial basis function neural network. The activation function for the each hidden unit in hidden layer for an input vector $x_{j}$ is calculated as per given Equation (9), where $\mu_{i}$ is the $i_{th}$ cluster center in the hidden layer and σ, also known as spread, was the smoothing parameter,

(9) $$g_{i}\left(x_{j}\right)=exp\left(\begin{array}{c} \frac{-\left|\right|x_{j}-\mu_{i}{\left|\right|}^{2}}{2\sigma_{i}^{2}} \end{array}\right),j=1,2,\ldots ,h$$

Following steps followed to determine the cluster center k-mean algorithm is utilized:

- **Step 1.** Initial set of cluster center was supplied to the hidden layer.
- **Step 2.** Then based on the Euclidean distance metrics used to assign the closest cluster center for each input of the hidden layer.
- **Step 3.** New cluster center computed $\mu_{k}$.
- **Step 4.** If the cluster center changed then repeat the step 2 and step 3; otherwise, stop.

The output layer neurons are fully connected to the hidden layer through the weights $w_{i}k$. The $k^{th}$ output value for an input $x_{j}$ is calculated as per Equation (10), where

(10) $$y_{k}\left(x_{j}\right)=\sum_{1}^{h}w_{ik}g_{i}\left(x_{j}\right),k=1,2,\ldots ,n$$

#### 4.5.5. Train, Validate, and Test RBFNN over Collected Data

**Figure 8:**
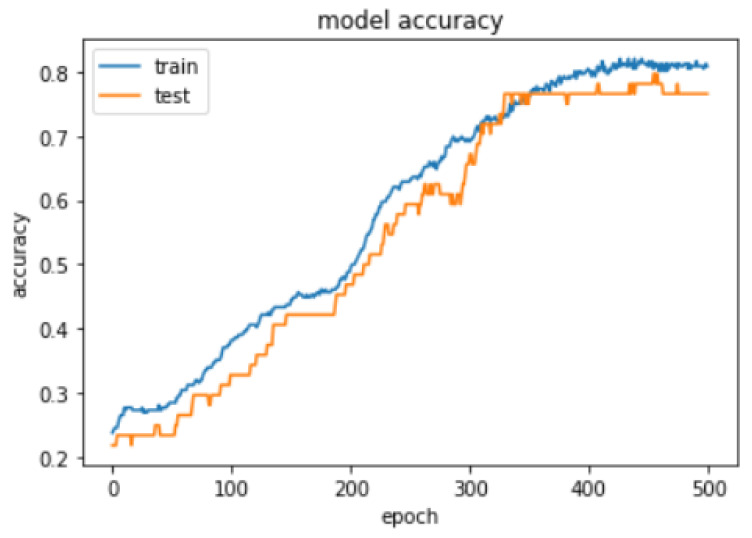
RBFNN accuracy curve.

**Figure 9:**
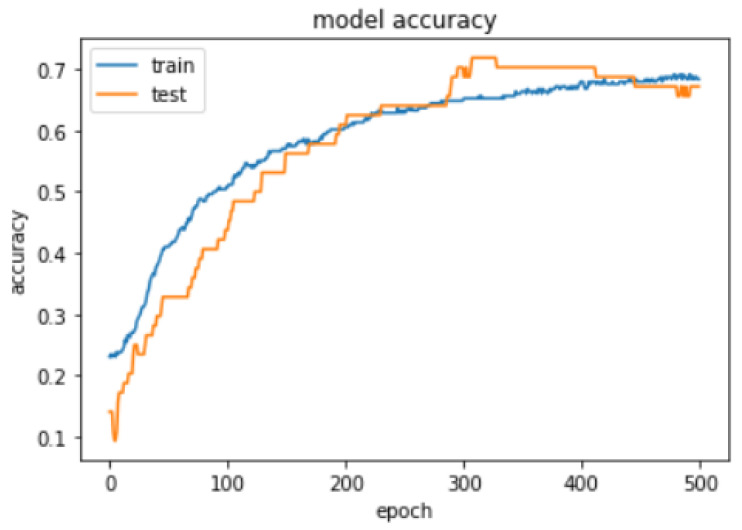
BPNN accuracy curve.

**Figure 10:**
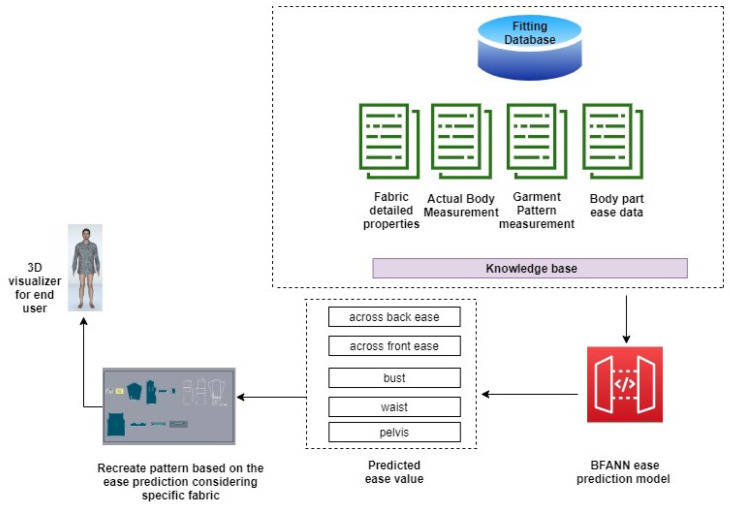
3D ease prediction service module.

1. Selection of σ value for the hidden layer has been identified by training the and validating model with 20% data, 500 epoch, beta value is 1, and momentum of RMSprop is 0.0, learning rate is 0.001 and 60 neurons in hidden layer with 12 different σ values as shown in Table 12. The model’s σ value insight has been taken based on the MSE value in the resultant table sigma value 0.1 to 1 has shown continuous decrease in the MSE values and gradual increase in the model’s accuracy after the sigma value 1 to 3 consistent decrease in the model’s accuracy and increase in the MSE value, except the sigma value 2.5 has shown slight increase but very low as compare to the 0 to 1 range of the sigma values. Therefore, σ=1 has been utilized to build the RBFNN model.
2. Comparison of the RBFNN model with Backpropagation neural network (BPNN) and the difference between the two models on the same data sample with 20% of the validation set; the accuracy curve for train and validation set is created. Accuracy curve data for 500 epochs for RBFNN and BPNN is shown in Table 13 and Table 14. Figure 8 and Figure 9 clearly show that the RBFNN model’s accuracy curve corresponding to test set has more generalized accuracy curve unlike the BPNN accuracy curve against the validation set. RBFNN and BPNN model compared over the 500 epochs and result can be seen in Table 15.
3. The application of ease prediction model has shown in Figure 10. Following sample record has been utilized to get the ease values for five key body parts of shirt across back, across front, bust, waist, pelvis. Input vector x = [34.71, 37.04, 86.98, 87.98, 73.89, 44.33, 44.69, 91.89, 103.88, 104.49, 95, 0.05, 5.89, 3.56, 2.85, 6.27, 0.76, 0.67, 5.3, 10.35, 0.81, 0.81, 14.72, 14.72] supplied to model and then resultant y vector returned y = [9.299779, 7.5186224, 4.83331, 15.186139, 30.181925], which represents ease of five key body parts across back, across front, bust, waist, pelvis. Further designers have utilized the predicted ease values to recreate the shirt pattern using the 3D CAD tool. This method gives quick insight by showing the variations in ease values due to the change in body measurement, fabric, and pattern size, without repeating the complete process in the 3D CAD tool.
4. Computational time for the ease prediction model after deploying it as a service is 45 ms.

All above steps have been organized as shown in Figure 3 for the creation of data-driven interactive design system.

## 5. Service Architecture Validation

**Figure 11:**
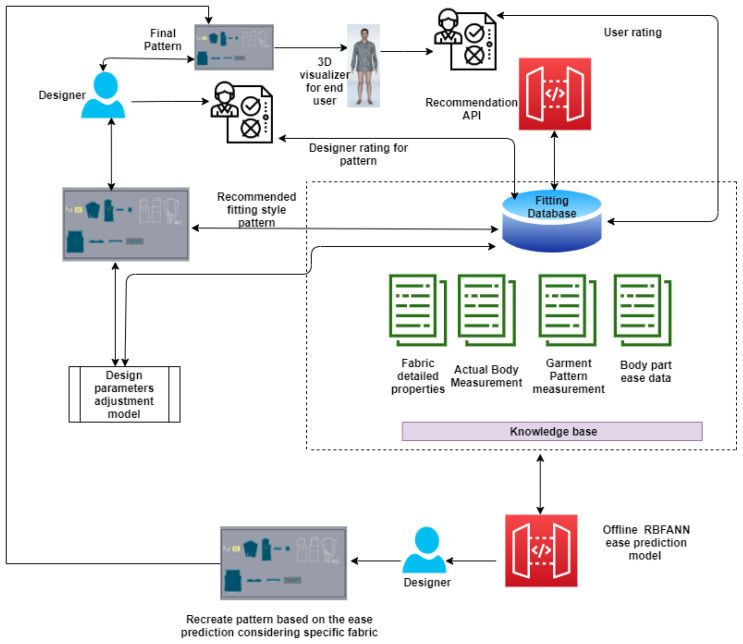
General architecture of the garment co-design interactive system.

This section puts light on the general architecture of the garment co-design interactive system Figure 11, which is utilized to evaluate and integrate the proposed data driven interactive design service architecture. Figure 11 shows the complete process of service integration with the offline designing tool and online e-shopping platform using the proposed data-driven service architecture. Following the architecture shown in Figure 11, a number of interactions can be realized between the consumer and the designer by repeatedly performing the cycle of design recommendation—virtual fitting visualization—interactive performance evaluation and selection—design parameters adjustment. The following points explains the changes, which will occur after following the proposed system structure.

1. The system creates a new BIRCH cluster object without affecting the old clustered data and should append the new data to the existing cluster, If new data do not form a new cluster.
2. The system introduces a new adaptive random forest classifier object, if a new fitting type is introduced in the data.
3. The system creates a frequent pattern table in the SQL Server database automatically corresponding to new fitting type, which may occur in new or old BIRCH cluster object.
4. The system creates association rule table in SQL server corresponding to new fitting, which may occur in new or old BIRCH cluster object.
5. The system integrates real fabric to existing knowledge base by parsing the fabric drape image, which is extracted from the drape meter and fabric drape image profile shall be created and saved into system.
6. Updated shirt pattern measurements, body measurements, ease allowance value of key body parts and fabric details shall be saved into SQL server database table schema to retrain the RBFNN ease prediction model.

An application case shall run following statements successfully to get the best design selection for users and designers from data driven interactive design system.

**Figure 12:**
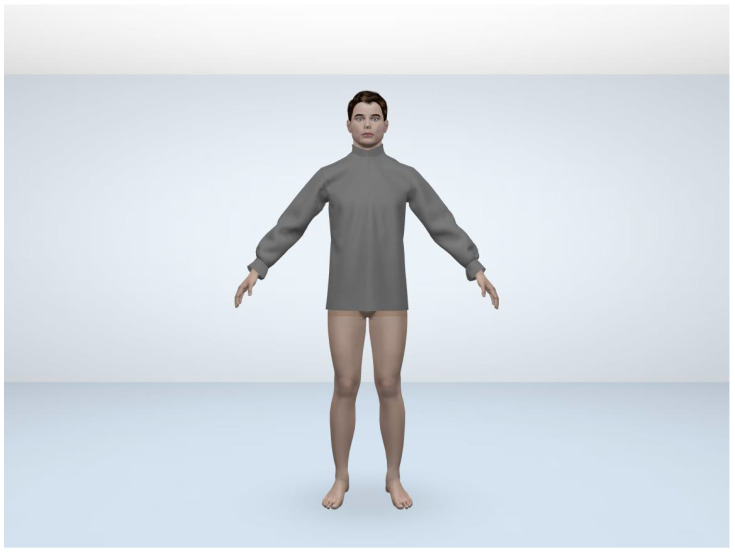
Shirt before adjustment.

**Figure 13:**
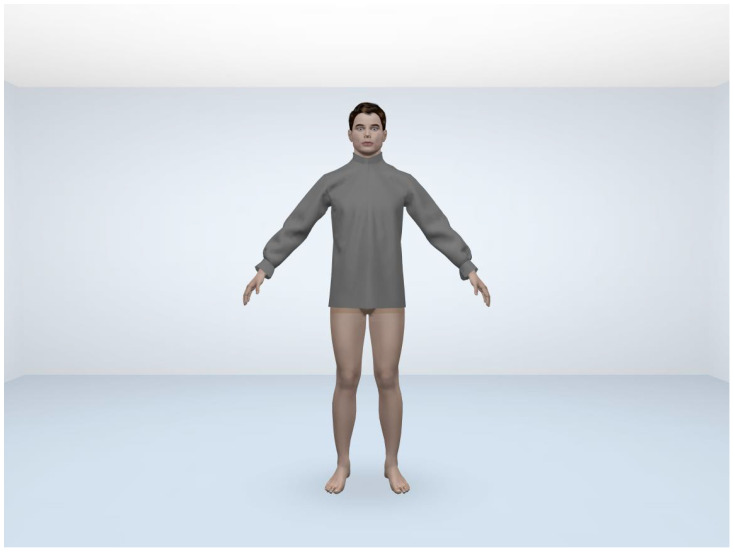
Shirt after adjustment.

**Figure 14:**
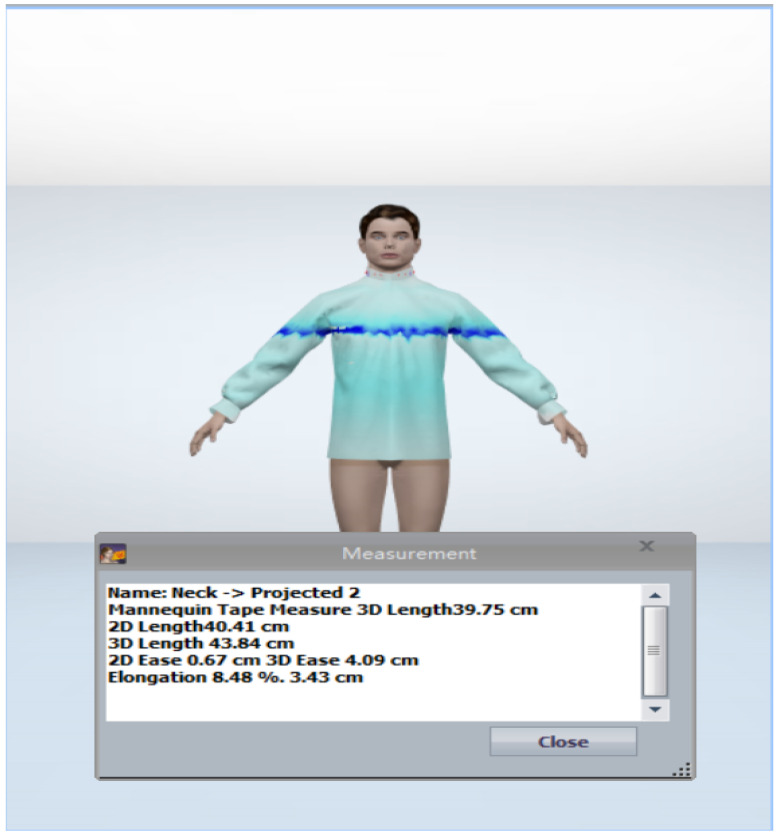
Shirt fitting before adjustment.

**Figure 15:**
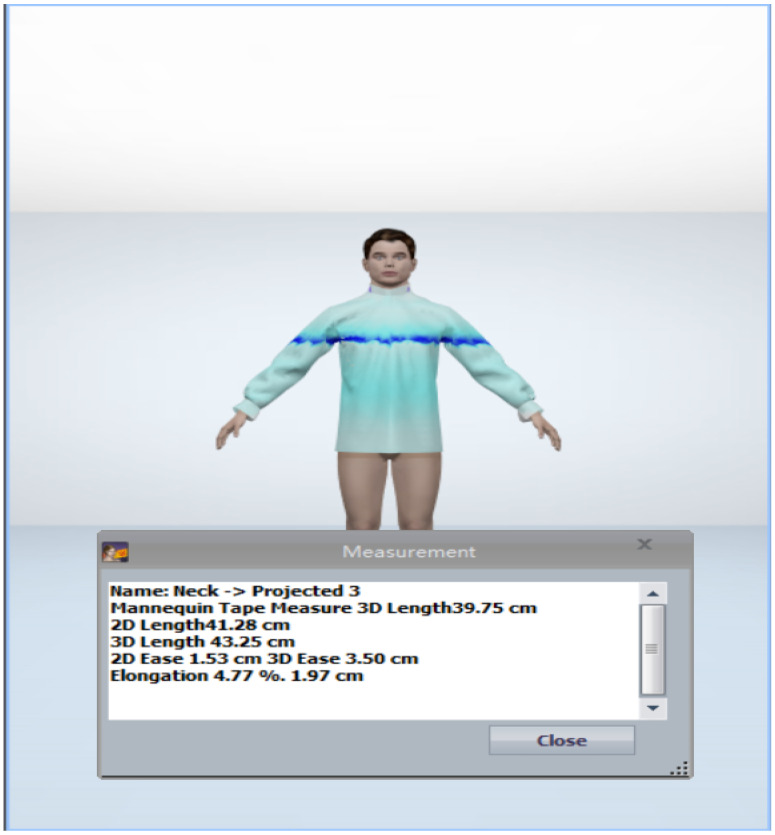
Shirt fitting after adjustment.

1. Most relevant biometric profile influenced group for a specific consumer.
2. Most relevant fitting style of shirt as per the biometric profile influenced group.
3. Identification of the relevant style attributes for predicted fitting style.
4. Identification of the closest fabric by comparing the drape.
5. Identification of the parametric value of garment ease corresponding to predicted styles of shirt. Then pattern creation and evaluation of the pattern by the designers.
6. In Section 4.3.2, point 7 set of the predicted values based on the user’s biometric profile has been carried further in Section 4.5.5, where point 3 is used to get the ease value for the key body parts specified by the professional designers. In this step, prediction values were generated using all the aforementioned five modules and has been carried further by designers to create the virtual garment in 3D CAD tool. The design solutions before and after the adjustment can be transformed into the 3D garment fitting effects and demonstrated in a virtual environment (see Figure 12 and Figure 13). In this case, the visual effects for these two design solutions are slight. However, we can see the difference by looking at the numerical parameters of the garment pressure images Figure 14 and Figure 15 that there is a difference at neck position. The neck length of the human model is 39.75 cm. The garment neck length is 43.84 cm before the adjustment and 43.25 cm after the adjustment (5 cm shorter). Its elongation value shows that the fabric has become a little tight. All these changes mean that the adjusted garment gives more fitting effects and closer to the wearer’s body surface. In the cases of tight style clothing such as legging, this change can be more evident.
7. Further, we have in-cooperated the Likert scale method having rating level up to five which helps in the understanding the garment ease satisfaction level (statistics for eight shirt patterns over four body measurements and one single fabric 100% cotton). We realized after the basic pattern adjustments as shown in Table 16 that 60% of customers come under the satisfied range of the scale, out of which 21% are highly satisfied, 31% are satisfied, and 47% have no problems with the garment ease of the given design pattern. Only 40% of customers come under the dissatisfaction range of the scale for the garment ease of the given design pattern. These results, in comparison with those in Table 17, clearly show that continuous data update can clearly improve the satisfaction rate on system’s results and system’s capacity to learn from updated data, which can be evaluated effectively.

## 6. Conclusions

In this paper, we have proposed a data-driven interactive design system architecture in order to help the designers to create the most relevant customized garment based on the garment buyer’s preferences in combination with designer’s technical knowledge. The proposed system architecture supports the process to formalize the structure of customized garment design process. Based on the designer’s feedback over the final results, it automatically adds the technicality on the insights generated for the garment buyers. Continuous data update mechanism and new data integration shows progressive enhancement in proposed system architecture. Proposed system can be utilized. In this context, the proposed system is directly involved in the process of increasing the proximity between designers, consumers, and fabric manufacturers. In future, service can be extended further by receiving more parametric information from 3D CAD garment design tools to build strong relationship between the fashion consumers and designers.

The following abbreviations are used in this manuscript:

## Figures and Tables

**Table 1:** Detailed description of clustered data.

Fit	Cluster_0	Cluster_1
Super Slim Fit	1112	136
Comfort fit	357	770
Regular	1752	771
Total	3221	1677

**Table 2:** Prequential evaluation of online adaptive random forest (ARF) classifier.

Mean Performance	Cluster 1	Cluster 2
arf—Accuracy	0.7298	0.7773
arf—Kappa	0.4405	0.587

**Table 3:** Frequent item set statistic for each fitting class.

Cluster 1		
Fitting type	FrequentItemset count	support
ComfortFit	16	10–87%
Regular	58	10–100%
SuperSlimFit	15	10–92%
**Cluster 2**		
ComfortFit	23	10–89%
Regular	58	10–100%
SuperSlimFit	19	10–89%

**Table 4:** Association rules statistic corresponding to each fitting class.

Cluster 1			
Fitting type	Number of Rules count	support	confidence
ComfortFit	5	11–46%	91–97%
Regular	39	11–50%	87–100%
SuperSlimFit	4	10–42%	93–96%
**Cluster 2**			
ComfortFit	7	10–27%	89–95%
Regular	35	12–35%	80–100%
SuperSlimFit	5	11–23%	90–100%

**Table 5:** Association rules for shirt feature cuff.

Id	Antecedent	Consequent	Confidence	Lift
1	{’Square 2 Buttons _cuff’}	{’Classic Point _collar’}	0.875	3.44
2	{’Square 2 Buttons _cuff’, ’15_735.2BENGO2_20_fabric’}	{’Classic Point _collar’}	0.875	3.44
3	{’Square 2 Buttons _cuff’}	{’Classic Point _collar’, ’15_735.2BENGO2_20_fabric’}	0.875	3.44
4	{’Italian Semi-Spread_collar’, ’Round Single_cuff’}	{’n_collarwhite’}	1	1.072
5	{’Italian Semi-Spread_collar’, ’15_735.2BENGO2_20_fabric’, ’Round Single_cuff’}	{’n_collarwhite’}	1	1.072
6	{’Italian Semi-Spread_collar’, ’Round Single_cuff’}	{’n_collarwhite’, ’15_735.2BENGO2_20_fabric’}	1	1.072
7	{’Hai Cutaway_collar’, ’Round Single_cuff’}	{’n_collarwhite’}	1	1.072
8	{’Hai Cutaway_collar’, ’15_735.2BENGO2_20_fabric’, ’Round Single_cuff’}	{’n_collarwhite’}	1	1.072
9	{’Hai Cutaway_collar’, ’Round Single_cuff’}	{’n_collarwhite’, ’15_735.2BENGO2_20_fabric’}	1	1.072
10	{’Classic Point _collar’, ’Round Single_cuff’}	{’n_collarwhite’}	1	1.072
11	{’Classic Point _collar’, ’15_735.2BENGO2_20_fabric’, ’Round Single_cuff’}	{’n_collarwhite’}	1	1.072
12	{’Classic Point _collar’, ’Round Single_cuff’}	{’n_collarwhite’, ’15_735.2BENGO2_20_fabric’}	1	1.072
13	{’Double inc. Cufflinks_cuff’}	{’n_collarwhite’}	1	1.072
14	{’Double inc. Cufflinks_cuff’, ’15_735.2BENGO2_20_fabric’}	{’n_collarwhite’}	1	1.072
15	{’Double inc. Cufflinks_cuff’}	{’n_collarwhite’, ’15_735.2BENGO2_20_fabric’}	1	1.072
16	{’Round Single_cuff’}	{’n_collarwhite’}	0.9677	1.038
17	{’15_735.2BENGO2_20_fabric’, ’Round Single_cuff’}	{’n_collarwhite’}	0.967	1.038
18	{’Round Single_cuff’}	{’n_collarwhite’, ’15_735.2BENGO2_20_fabric’}	0.967	1.038
19	{’Classic Point_collar’, ’Round Single_cuff’}	{’n_collarwhite’}	0.95	1.023
20	{’Double inc. Cufflinks_cuff’}	{’n_collarwhite’}	0.94	1.014
21	{’Round Single_cuff’}	{’n_collarwhite’}	0.93	1.00

**Table 6:** 3D Mannequins key body part’s measurement data for virtual shirt.

3D Mannequin’s Key Body Measurement for Shirt					
**No**	**Across Back**	**Across Front**	**Bust**	**Waist**	**Pelvis**
1	34.71	37.04	86.98	87.98	73.89
2	35.71	38.11	90.99	91.87	78
3	36.73	39.85	95	95.96	82.01
4	37.75	40.3	99.01	99.99	86.02
5	38.86	41.49	103	104	90.04
6	39.29	41.95	107	107.83	94
7	40.67	43.42	110.99	111.41	98.01
8	41.47	44.29	114.97	115	102.21

**Table 7:** Standard men’s shirt size chart.

Id	Size	Size Unit	Length	Breat Girth	Sleeve Length	Sleeve Opening
1	38	cm	62.00	82.00	63.00	16.00
2	40	cm	64.00	86.00	64.00	17.20
3	42	cm	66.00	90.00	65.00	18.40
4	44	cm	68.00	94.00	66.00	19.60
5	46	cm	70.00	98.00	67.00	20.80
6	48	cm	72.00	102.00	68.00	22.00
7	50	cm	74.00	106.00	69.00	23.20
8	52	cm	76.00	110.00	70.00	24.40

**Table 8:** Pattern measurement for key body part’s of shirt.

Key Body Parts Pattern Measurements for Shirt					
**Across Back**	**Across Front**	**Bust**	**Waist**	**Pelvis**	**Size**
44.33	44.69	91.89	103.88	104.49	38
44.93	46.03	96.54	107.6	109.12	40
46	47.46	96.47	111.29	112.39	42
47.11	48.09	98.31	115.07	116.27	44
48.36	49.53	100.12	118.82	120.42	46
49.48	50.65	102.02	122.87	122.96	48
50.71	51.49	104.4	126.2	127.16	50
52.09	52.59	107.78	130.53	131.09	52

**Table 9:** Fabric data with mechanical properties.

Id	W	C	TH	BR1	BR2	TREMT1	TREMT2	TRLT1
1	95.00	100% Cotton	0.05	5.89	3.56	2.85	6.27	0.76
2	288.00	100% Wool	0.07	17.66	11.77	5.80	3.94	0.69
3	32.00	100% Silk	0.05	0.74	0.34	4.35	16.70	0.80
4	342.00	100% Cotton	0.05	34.64	21.46	9.23	5.18	0.69
5	192.00	94% Cotton 5% Polymid 1% Elasthane	0.06	9.93	8.71	4.73	27.23	0.65

**Table 10:** Continuation of Table 9.

Id	TRLT2	TRWT1	TRWT2	SRG1	SRG2	SRT1	SRT2
1	0.67	5.30	10.35	0.81	0.81	14.72	14.72
2	0.70	9.76	6.77	1.72	1.53	14.72	14.72
3	0.81	0.85	3.33	0.17	0.17	9.81	9.81
4	0.66	15.65	8.39	3.37	3.01	49.05	49.05
5	0.53	7.51	36.94	1.84	1.35	15.00	15.00

**Table 11:** Fabric properties description table for Table 9 and Table 10.

Abbreviation	Description	Abbreviation	Description
**W**	Weight	**TR** **LT2**	TensileResistanceLTWeft
**C**	Composition	**TR** **WT1**	TensileResistanceWTWarp
**Th**	Thickness (unit cm)	**TR** **WT2**	TensileResistanceWTWeft
**BR1**	BendingResistanceWarp	**SR** **G1**	ShearingResistanceGWarp
**BR2**	BendingResistanceWeft	**SR** **G2**	ShearingResistanceGWeft
**TR** **EMT1**	TensileResistanceEMTWarp	**SR** **T1**	ShearingResistanceTWarp
**TR** **EMT2**	TensileResistanceEMTWeft	**SR** **T2**	ShearingResistanceTWeft
**TR** **LT1**	TensileResistanceLTWarp		

**Table 12:** Calculated and analyzed MSE for the different sigma values.

Epoch	Sigma	Mse	Accuracy
500	0.1	0.0188	0.7578
500	0.25	0.0079	0.793
500	0.5	0.0058	0.793
500	1	0.0028	0.8047
500	1.25	0.0027	0.7969
500	1.5	0.0070	0.7617
500	1.75	0.0153	0.7383
500	2	0.0418	0.6562
500	2.25	0.1161	0.4883
500	2.5	0.2044	0.5234
500	2.75	0.3204	0.4531
500	3	0.3391	0.3711

**Table 13:** RBFNN accuracy curve.

Epoch	Loss	Accuracy	Mse	Val_loss	Val_accuracy	Val_mse
0	1.025581	0.191406	1.025581	0.876186	0.171875	0.876186
1	1.020244	0.195312	1.020244	0.873400	0.171875	0.873400
2	1.016299	0.199219	1.016299	0.870802	0.171875	0.870802
3	1.012945	0.199219	1.012945	0.868433	0.171875	0.868433
4	1.009710	0.207031	1.009710	0.866253	0.171875	0.866253
..	...	...	...	...	...	...
495	0.002823	0.808594	0.002823	0.012917	0.765625	0.012917
496	0.002815	0.804688	0.002815	0.012571	0.781250	0.012571
497	0.002913	0.808594	0.002913	0.012797	0.765625	0.012797
498	0.002861	0.804688	0.002861	0.012664	0.765625	0.012664
499	0.002823	0.804688	0.002823	0.012685	0.781250	0.012685

**Table 14:** BPNN accuracy curve.

BPNN						
**Epoch**	**Loss**	**Accuracy**	**Mse**	**Val_loss**	**Val_accuracy**	**Val_mse**
0	1.255189	0.230469	1.255189	0.973620	0.140625	0.973620
1	1.065259	0.234375	1.065259	0.823501	0.140625	0.823501
2	0.909692	0.230469	0.909692	0.702508	0.140625	0.702508
3	0.780366	0.234375	0.780366	0.598431	0.109375	0.598431
4	0.669734	0.234375	0.669734	0.511944	0.093750	0.511944
..	...	...	**...**	...	**...**	**...**
495	0.005829	0.683594	0.005829	0.006387	0.671875	0.006387
496	0.005823	0.687500	0.005823	0.006382	0.671875	0.006382
497	0.005820	0.687500	0.005820	0.006380	0.671875	0.006380
498	0.005809	0.683594	0.005809	0.006372	0.671875	0.006372
499	0.005803	0.683594	0.005803	0.006365	0.671875	0.006365

**Table 15:** RBFNN and BPNN comparison.

RBFNN						
**Epoch**	**Loss**	**Accuracy**	**Mse**	**Val_loss**	**Val_accuracy**	**Val_mse**
499	0.002823	0.804688	0.002823	0.012685	0.781250	0.012685
**BPNN**						
**Epoch**	**Loss**	**Accuracy**	**Mse**	**Val_loss**	**Val_accuracy**	**Val_mse**
499	0.005803	0.683594	0.005803	0.006365	0.671875	0.006365

**Table 16:** Satisfaction Levels for regular fitting design patterns (after adjustment).

Average High Satisfaction Range	60%	Average Lower Satisfaction Range	40%
Extremely satisfied	21%	Slightly satisfied	69%
Very satisfied	31%	Not at all satisfied	30%
Satisfied	41%		

**Table 17:** Satisfaction Levels for regular fitting design patterns (before adjustment).

Average High Satisfaction Range	53%	Average Lower Satisfaction Range	46%
Extremely satisfied	35%	Slightly satisfied	40%
Very satisfied	30%	Not at all satisfied	60%
Satisfied	35%		

## Data Availability

Not applicable.
